# Comparison of the cardiovascular presentations, complications and outcomes following different coronaviruses’ infection: A systematic review

**DOI:** 10.34172/jcvtr.2021.29

**Published:** 2021-04-24

**Authors:** Mohammad Mostafa Ansari Ramandi, Hossein Yarmohammadi, Somayeh Beikmohammadi, Behzad Hassan Hosseiny Fahimi, Farbod Hatami, Hossein Beydokhti, Hooman Bakhshandeh, Nasim Naderi

**Affiliations:** ^1^Cardiovascular Diseases Research Center, Birjand University of Medical Sciences, Birjand, Iran; ^2^Medical Students Research Committee, Shahed University, Tehran, Iran; ^3^Department of Cardiology, Universitätsmedizin Schleswig-Holstein, Lubeck, Germany; ^4^Faculty of Medicine, Birjand University of Medical Sciences, Birjand, Iran; ^5^Rajaie Cardiovascular Medical and Research Center, Iran University of Medical Sciences, Tehran, Iran

**Keywords:** COVID-19, SARS, MERS, Cardiovascular, Manifestation

## Abstract

Manifestations caused by coronavirus family have presented it in many ways during the previous years. The aim of this systematic review was to gather all possible cardiovascular manifestations of the coronavirus family in the literature. Adhering to Preferred Reporting Items for Systematic Reviews and Meta-Analyses (PRISMA) guidelines, we searched PubMed, Scopus, Web of Science, Cochrane and ProQuest which were updated on May 1, 2020 for the last time. Regarding to the novelty and speed of publications on COVID-19, we searched Google Scholar and also references of included studies and review articles in the systematic search results were searched manually. The searched keywords were the combination of the following MeSH terms: "COVID-19", "SARS", "MERS" and "cardiovascular presentation". The systematic review was registered with ID CRD42020180736 in International Prospective Register of Systematic Reviews (PROSPERO). After screening, 28 original articles and ten case studies (five case reports and five case series) were included. Most of the studies were focused on COVID-19 (20 original articles and four case studies) while the only studies about Middle East Respiratory Syndrome (MERS) were a case report and a case series. Almost all the cardiovascular presentations and complications including acute cardiac injury, arrhythmias and the thrombotic complications were more prevalent in COVID-19 than severe acute respiratory syndrome (SARS) and MERS. The cardiac injury was the most common cardiovascular presentation and complication in COVID-19 whereas thrombotic complications were commonly reported in SARS. The cardiac injury was the predictor of disease severity and mortality in both COVID-19 and SARS.Coronavirus 2019 may present with cardiovascular manifestations and complications in signs and symptoms, laboratory data and other paraclinical findings. Also, cardiovascular complications in the course of COVID-19 may result in worse outcomes.

## Introduction

The novel coronavirus disease 2019 (COVID-19) pandemic has rapidly spread in many countries around the world. As of June 18, 2020, more than 8 million people have been infected, with near to 440 000 deaths.^1^ COVID-19 is affecting adults more than children specially those younger than 15 years of age. The three main symptoms of the infection are cough, shortness of breath and fever. Some other symptoms include headache, malaise and sore throat which are less common. Most of these symptoms are presentations of respiratory tract infection. However, there have been reports about symptoms which overlap with cardiovascular symptoms.^2^

Some of these patients have underlying cardiovascular diseases, which affects their disease progression and outcome, but others might also present with cardiovascular manifestations. They might either present with cardiovascular findings or develop cardiovascular complication.^2,3^ But this is not the first time for cardiovascular presentations of coronavirus. There have been some studies reporting these presentations by previously known strains of the coronavirus family. Severe acute respiratory syndrome (SARS) caused by SARS-associated coronavirus has presented itself in many ways during the previous years.^4-7^

Studying the previous presentations of coronavirus family and the recent cardiovascular manifestations of COVID-19 can help in predicting possible future challenges and taking measures to tackle these issues. The aim of this systematic review was to gather cardiovascular presentations of the coronavirus diseases including SARS, MERS and COVID-19 in the literature.

## Material and Methods

### Search strategy

PubMed, Scopus, Web of Science, Cochrane and ProQuest were searched systematically based on the Preferred Reporting Items for Systematic Reviews and Meta-Analyses (PRISMA) guideline and updated on May 1, 2020 for the last time.^8^ Regarding to the novelty and speed of publications on COVID-19, we searched Google Scholar and also references of included studies and review articles in the systematic search results were searched manually. The searched keywords were the combination of the following MeSH terms: “COVID-19”, “SARS”, “MERS” and “cardiovascular presentation”. The systematic review was registered with ID CRD42020180736 in PROSPERO International prospective register of systematic reviews and the complete search strategy is available online. On May 6, 2020, we added another researcher who helped in carrying on the systematic review to the PROSPERO. We also added the search strategy for all databases that were used. There were no changes in the protocol during the review.

### Study selection

Interventional or descriptive studies on cardiovascular manifestations or complications of confirmed cases of three Coronaviruses including COVID-19, MERS and SARS were included in the study. There was no limitation on the year of publication or age of the study population. However, only articles in English Language were included. Case reports and letters were not excluded. Animal studies, reviews and guidelines were considered as exclusion criteria. Title, abstract and full text of search results were screened by two investigators independently and in any case of disagreement, the third senior investigator was asked to make the decision after discussion about the issue.

Our outcomes of interest were the various cardiovascular specific presentations, the cardiovascular related laboratory and imaging findings, complications and outcomes in SARS, MERS and COVID-19.

### Data extraction

For each included study, the type of Coronavirus infection, year and country of publication, and the name of the first author were summarized into a table. Reported cardiac presentations and complications with their prevalence in the study population if available, were gathered and their impact on outcomes of the disease were added to the table. The data extraction was performed by two investigators independently and the third investigator was asked to participate in the case of disagreement.

### Quality assessment

For quality assessment, the Quality Assessment Tool for Studies with Diverse Designs (QATSDD) was used.^9^ Two investigators assessed each study independently and the third investigator was asked to make the decision in the case of disagreement. The quality assessment was only done for original articles and case studies were not assessed. Also, quality assessment was not done for excluded articles. The QATSDD consists of 16 questions that 2 of them are for qualitative studies only. For each question there is a possible score 0 to 3 that makes the highest possible score to be 42. After scoring each question, the overall score for each article was calculated and then the percentage of the 42 was determined.

## Results

**Figure 1:**
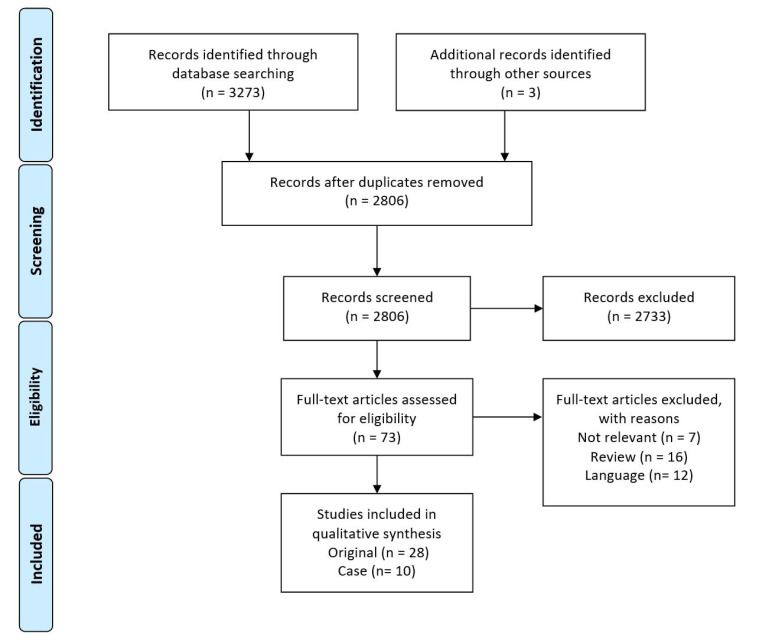
PRISMA flow diagram

After screening, 28 original articles and 10 case studies (5 case reports and 5 case series) were included. Most of the studies were focused on COVID-19 (20 original articles and four case studies), and 12 articles (eight original and four case studies) were focused on SARS. While there were only two studies about MERS. The PRISMA flow diagram is shown in Figure 1. Quality of most 20 studies was more than 70%. The range of the percent of quality was 40% to 88%. Original studies were retrospective in 22 articles and China was the origin of most of the articles.

The original articles and case reports are summarized in Table 1 and Table 2 respectively.

**Table 1:** Included original studies in the manuscript

**Coronavirus family**	**Author**	**Place, year**	**Design (No)**	**Cardiovascular presentations, No (percentage)**	**Cardiovascular complications, No (percentage)**	**Cardiovascular outcome, No (percentage)**	**Quality (%)**
COVID-19	Guo et al.^10^	China, 2020	Retrospective, single center (187)	Ÿ Myocardial injury 52 (27.8%) Ÿ VT/VF 11 (5.9%)	NA	Patients with myocardial injury had: More males Higher age Shorter duration of symptoms to death or discharge Higher mortality More complications	71
	Wan et al.^11^	China, 2020	Retrospective, single center (135)	NA	Ÿ Acute cardiac injury, 10 (7.4%)	Patients with higher CK MB: Ÿ Were more severe case	76
	Huang et al.^12^	China, 2020	Prospective, multicenter (41)	NA	Ÿ Acute cardiac injury 5 (12%)	Patients with acute cardiac injury had: Ÿ More need for ICU care.	83
	Zhou et al.^13^	China, 2020	Retrospective, multicenter (191)	NA	Ÿ Acute cardiac injury, 33 (17%) Ÿ Heart failure, 44 (23%)	Patients with acute cardiac injury or heart failure had: Ÿ Higher mortality	78
	Chen et al.^14^	China, 2020	Retrospective, single center (274)	Ÿ HscTnI >15.6 pg/mL, 83/203 (41%) Ÿ NT-proBNP ≥285 pg/mL, 85/173 (49%)	Ÿ Acute cardiac injury, 72/94 (77%) Ÿ Heart failure, 41/83 (49%)	Patients with cardiovascular complications had: Ÿ Higher mortality	78
	Du et al.^15^	China, 2020	Retrospective, multicenter (85)	Ÿ CK>170U/L, 31 (36.5%)	Ÿ Acute cardiac injury, 38 (44.7%) Ÿ Arrhythmia 51 (60%)	Cause of death: Ÿ Cardiac arrest, 7/81 (8.64%) Ÿ Acute coronary syndrome, 4/81 (4.94%) Ÿ Malignant arrhythmia, 2/81 (2.47%)	69
	Chen et al.^15^	China, 2020	Experimental study on human heart tissue, single center (45)	Ÿ SARS-CoV-2 might attack pericytes, and cause capillary endothelial cells dysfunction	NA	NA	42
	Guan et al.^16^	China, 2019	Retrospective, multicenter (1099)	Ÿ CK ≥ 200 U/l, 90/657 (13.7 %)	NA	NA	85
	Zhang et al.^17^	China, 2020	Retrospective, single center (140)	Ÿ Increased serum CK, 4/60 (6.7%)	NA	NA	71
	Wang et al.^18^	China, 2020	Retrospective, single center (339)		Acute cardiac injury, 70 (21.0%) Ÿ Arrhythmia, 35 (10.4 %) Ÿ Cardiac insufficiency, 58 (17.4%)	Higher mortality was seen in patients with: Acute cardiac injury Ÿ Arrhythmia Ÿ Cardiac insufficiency	83
	Shi et al.^19^	China, 2020	Retrospective observational cohort, single center (416)	Ÿ 14 abnormal ECGs in cardiac injury patients: myocardial ischemia, T-wave depression and inversion, ST-segment depression, and Q waves.	NA	Higher mortality was seen in patients with: Cardiac injury. More than half of the patients with cardiac injury experienced in-hospital death in this study, indicating that COVID-19–induced cardiac injury is associated with major adverse clinical outcomes.	88
	Chen et al.^20^	China, 2020	Retrospective, single center (99)	Ÿ Elevated CK, 13 (13%)	Ÿ One patient developed severe respiratory failure, heart failure, and sepsis. Expired due to sudden cardiac arrest on the 11th day of admission.	NA	69
	Yang et al.^21^	China, 2020	Retrospective, single center (52)	Ÿ Cardiac injury, 12 (23%)	NA	NA	54
	Bonetti et al.^22^	Italy, 2020	Retrospective, single center (144)	NA	NA	Non- survivors had higher values of: Ÿ CK. Ÿ hscTnI.	71
	Zheng et al.^23^	China. 2020	Retrospective, single center (161)	Ÿ CK ≥ 190 U/l, 17 (10.6 %)	NA	Patients with high CK had more: Ÿ Severe disease	76
	Han et al.^24^	China, 2020	Retrospective, single center (273)	Ÿ CK-MB > 5 ng/ml, 10 (3.66%) ŸUltra-TnI > 0.04 ng/ml, 27 (9.89%) ŸNT-proBNP > 900 pg/mL, 34 (12.45%)	NA	Severity and case-fatality rate of COVID-19 was associated with: Ÿ Higher concentration of CK-MB Ÿ Higher concentration of ultra-TnI Ÿ Higher concentration of NT-proBNP	76
	Yang et al.^25^	China, 2020	Retrospective, single center (92)	NA	Ÿ Cardiac Injury, 31/91 (34.1%)	Cause of death: Ÿ AMI, 6 (6.5%) Ÿ Heart failure 2 (2.2%)	47
	Deng et al.^26^	China, 2020	Retrospective single center (112)	Ÿ LVEF <50%, 6 (5.4%) Ÿ Possible myocarditis, 14 (12.5%) Ÿ Tachycardia, 33 (29.5%) Ÿ ST segment elevation/ST-T changes, 22 (19.6%) Ultra-TnI > 0.04 ng/ml, 42 (37.5%)	Ÿ NA	Ÿ For all the patients who died during hospitalization, cardiac markers were elevated before death and cardiac troponin I was peaked within a week preceding death. Ÿ Those with higher peak cardiac troponin and NT-pro BNP level had higher mortality.	78
	Hong et al.^27^	South Korea, 2020	Retrospective, single center (98)	NA	Ÿ Acute cardiac injury, 11 (11.2%)	Patients needing ICU care had more: Ÿ Acute Cardiac injury	69
	Zheng et al.^28^	China, 2020	Retrospective, single center (99)	NA	Ÿ NA	Critically ill patients had higher: Ÿ CK-MB Ÿ NT-proBNP Ÿ TnT	40
	**Coronavirus family**	**Author**	**Place, year**	**Design (No)**	**Cardiovascular presentations, No (percentage)**	**Cardiovascular complications, No (percentage)**	**Cardiovascular outcome, No (percentage)**	**Quality (%)**
SARS	Siu-lung li et al.^5^	Hong Kong, 2003	Prospective, single center (46)	Ÿ RBBB in ECG, 7 (15.2%) Ÿ Trace to mild mitral regurgitation, 17 (36.9%) Ÿ Trace to mild aortic regurgitation, 2 (4.3%) Ÿ Significantly higher left ventricular IMP at baseline vs day 30 (0.42 versus 0.33) Ÿ Longer IVRT at baseline vs day 30 (102.9 versus 81.6ms) Ÿ Lower FPV at baseline vs day 30 (69.6 versus 83.8 cm/s) Ÿ Lower doppler-derived CO at baseline vs day 30 (4.69 versus 5.49 L/min) Ÿ Lower Em at base line vs day 30 (17.3 versus 19.3 cm/s)	NA	Patients requiring mechanical ventilation had: Ÿ Lower LVEF at baseline Ÿ Higher IMP at baseline	83
	Yu et al.^29^	Hong Kong, 2006	Prospective, single center (121)	Ÿ Palpitation, 5 (4%) Ÿ Elevated CK, 31 (26%) Ÿ No significant increase in troponin or CK-MB Ÿ Tachycardia, 87 (71.9%) Ÿ Significant sinus bradycardia, 18 (14.9%)	Ÿ Transient paroxysmal atrial fibrillation on day 8 hospitalisation, lasted for 1 day and subsided spontaneously without treatment, 1 (0.82%) Ÿ Significant hypotension during the hospitalisation period, 61 (50.4%) Ÿ Cardiomegaly in first week of hospitalization, 8(6.6%) Ÿ Cardiomegaly in second week of hospitalization, 7(5.8%), Ÿ Cardiomegaly in third week of hospitalization, 4 (3.3%)	NA	85
	Gu et al.^30^	China, 2005	Retrospective, multi center, (18)	Ÿ No obvious pathologic change in the heart. Lymphocytes and monocytes were found in some of these organs, mostly within vessels	NA	NA	54
	Booth et al.^31^	Canada, 2003	Retrospective, multicenter (144)	Ÿ Tachycardia ŸAbnormal CK, 43/109 (39%)	ŸAbnormal CK, 64/118 (54%)	Ÿ Increased CK was significantly associated with poor outcome.	71
	Chan et al.^32^	China, 2003	Prospective, single centre (115)	NA	NA	Ÿ 2 patients died because of AMI Ÿ Increased CK did not affect mortality.	85
	Choi et al.^33^	Hong Kong, 2003	Retrospective cohort, single center. (267)	Ÿ CK was not elevated significantly in confirmed SARS cases.		Ÿ Post mortem analysis of 2 bodies revealed pulmonary thromboembolism as a cause for one of the deaths.	83
	Lee et al.^34^	Hong Kong, 2003	Prospective, single center (138)	ŸElevated CK levels, 44 (32.1%)	**NA**	Ÿ None of the patients with elevated CK levels had abnormal values for CK-MB or troponin T, indicating that the source of CK was unlikely to be cardiac muscle.	76
	Lew et al.^35^	Singapore, 2003	Retrospective, single center (199)	**NA**	Ÿ Ischemic stroke, 4 (2.01%) Ÿ DVT, 11 (5.52%) Ÿ Pulmonary embolism, 7 (3.51%)	Early Cause of death: Ÿ DCM, 1 (0.50%) Ÿ Cardiac failure with septicaemia shock, 1 (0.50%) Ÿ Ventricular fibrillation and end stage renal failure, 1 (0.50%) Late (>7 days) cause of death: ŸAMI, 1 (0.50%)	83

Abbreviations: AMI, acute myocardial infarction; CK-MB, Creatine Kinase MB; CO, cardiac output; COVID-19, Coronavirus Disease 2019; DCM, dilated cardiomyopathy; DVT, deep vein thrombosis; ECG, electrocardiography; FPV, flow propagation velocity; HscTnI, high sensitivity troponin I; ICU, Intensive Care Unit; IMP, myocardial performance index; IVRT, Isovolumetric relaxation time; NA, not available; No, number; NT-proBNP, N-terminal pro-brain natriuretic peptide; RBBB, right bundle branch block; SARS-CoV-2, severe acute respiratory syndrome coronavirus 2; VF, ventricular fibrillation; VT, ventricular tachycardia

**Table 2:** Included case studies in the manuscript

**Coronavirus family**	**Author**	**Place, year**	**Design (No)**	**Presenting history, sign and symptoms**	**Para-clinical evaluation**	**Outcome**
COVID-19	Fried et al.^3^	USA, 2020	Case series (4)	**Case 1:** 64 year-old female with hypertension and hyperlipidemia. Persistent chest pressure for two days. No remarkable sign on physical examination. **Case 2:** 38 year-old male with type 2 diabetes mellitus. One week of cough, pleuritic chest pain and progressive shortness of breath. Tachypnea on physical examination. **Case 3:** 64 year-old female with non-ischemic cardiomyopathy (recent normalization of LVEF), atrial fibrillation, hypertension and diabetes. Non-productive cough and shortness of breath for two days. **B**lood pressure 153/120 mmHg, heart rate 100 bpm, and oxygen saturation 88%. **Case 4:** A 51 year-old man with heart transplantation in 2007 and renal transplantation in 2010. Intermittent fever, dry cough, and shortness of breath for nine days.	**Case 1:** ECG: Sinus tachycardia at 102 bpm, low voltage QRS complexes in the limb leads, ST segment elevations in leads I, II, aVL, V2-V6 and PR elevation and ST depressions in aVR. Laboratory: Troponin I on admission was 7.9 ng/mL. Angiography: Non-obstructive coronary artery disease. Right heart catheterization: Consistent with cardiogenic shock. TTE: EF 30%. **Case 2:** Laboratory: High sensitivity troponin T was 1341 ng/L. TTE: Normal EFà20-25%, with akinesis of the mid left ventricular segments, and normal right ventricular size with mildly reduced function during admission **Case 3:** ECG: sinus rhythm, an isolated premature ventricular complex, premature atrial complexes, lateral T wave inversions and QTc 528 ms. Laboratory: NT-proBNP 6,137 pg/mL, HscTnT 42 ng/mL. TTE: Severe LV systolic dysfunction. Bedside pulmonary artery catheterization revealed a right atrial pressure of 10 mmHg, pulmonary artery pressure 45/20 mmHg, with a Fick cardiac index of 1.7 L/min/m2. **Case 4:** ECG: Normal sinus rhythm with new nonspecific T-wave inversions in the inferior and lateral leads. Laboratory: NT-proBNP 3212 pg/mL and HscTnT 16 ng/L. TTE: Normal cardiac allograft function.	**Case 1:** On IABP and dobutamine infusion, her cardiac index and lactate normalized and her end-organ function remained stable. The troponin-I peaked at 18.6 ng/mL and subsequently trended down to 0.4 ng/mL. The IABP was weaned after 7 days and the patient remained hemodynamically stable off IABP and inotropes. On repeat echocardiography on hospital day 10, LVEF improved to 50% and wall thickness was reduced. **Case:2** He developed a supraventricular tachycardia and was successfully cardioverted. Developed acidosis and low o2 saturation. VV ECMO. Vasopressor due to low blood pressure. VAV ECMO. Decannulated from ECMO after 7 days and is hemodynamically stable, although he remains on mechanical ventilation. **Case 3:** Respiratory status worsened rapidly, requiring intubation. She developed hypotension and was started on vasopressors. Dobutamine was started but was discontinued when she developed polymorphic ventricular tachycardia requiring cardioversion. IABP was considered but deferred due to improvement in the arterial lactate and blood pressure. Troponin levels remained relatively stable throughout (peak 214 ng/mL). She remains intubated on day 9 of her hospitalization due to agitation with ventilator weaning attempts. **Case 4:** Following admission, the mycophenolate mofetil was discontinued. Through the first five days of the hospitalization, the patient was intermittently febrile and his inflammatory markers remained persistently elevated, though he remained clinically stable. He was discharged home after 7 days in the hospital.
	Hu et al.^36^	China, 2020	Case report	A 37-year-old male. Chest pain and dyspnoea and diarrhoea for 3 days. Blood pressure: 80/50 mmHg.	Chest x ray: Significant enlargement of the heart. Chest CT: Pulmonary infection, enlarged heart, and pleural effusion. ECG: ST-segment elevation acute inferior myocardial infarction. CT coronary angiography: No coronary stenosis. Laboratory: Troponin T was more than 10 000ng/L. CK-MB 112.9ng/L. Natriuretic peptide BNP was up to 21 025ng/L. TTE: Enlarged heart and EF 27%, trace 2mm pericardial effusion.	Methylprednisolone (200mg/day, 4days). Immunoglobulin (20g/day, 4days). Norepinephrine. Furosemide. Milrinone. Symptoms improved significantly. One week later, normal heart size on chest x ray. Echocardiography showed that the size and function of the heart had returned to normal. Markers of myocardial injury dropped significantly after 1 week of treatment. After 3 weeks, the myocardial injury markers had fully recovered to the normal range.
	Inciardi et al.^37^	Italy, 2020	Case report	A healthy 53-year-old woman. Severe fatigue for 2 previous days. She denied chest pain, dyspnea, and further symptoms. She reported having fever and cough the week before. Blood pressure of 90/50 mmHg, heart rate of 100 beats per minute, oxygen saturation of 98% while breathing ambient air, and body temperature of 36.6 °C.	ECG: Low voltage in the limb leads, minimal diffuse ST-segment elevation, and an ST-segment depression with T-wave inversion in lead V1 and aVR. Chest x ray: Unremarkable. Laboratory: Elevated high-sensitivity troponin T level of 0.24 ng/mL and CK–MB level of 20.3ng/mL, elevated NT-pro BNP levels (5647pg/mL). TTE: RWMA with EF 40%. Circumferential pericardial effusion that was most notable around the right cardiac chambers (maximum, 11mm) without signs of tamponade Coronary angiography: No obstructive CAD. CMR: Confirmed the increased wall thickness with diffuse biventricular hypokinesis, especially in the apical segments,and severe LV dysfunction (LVEF of 35%). T2-mapping sequences showed marked biventricular myocardial interstitial diffuse late gadolinium enhancement. The myocardiale demand pattern of late gadolinium enhancement fulfilled all the Lake Louise criteria for the diagnosis of acute myocarditis.	Low BP. Dobutamine. TTE performed on day 6, revealed a significant reduction of LV wall thickness (interventricular septum, 11mm and posterior wall, 10mm), and improvement of LVEF to 44%, and a slight decrease of pericardial effusion (maximum, 8-9mm). At the time of submission the patient was hospitalized with progressive clinical and hemodynamic improvement.
		Xu et al.^38^	China, 2020	Case report	A 50-year-old man. Symptoms of fever, chills, cough, fatigue and shortness of breath.	Chest x-ray: showed multiple patchy shadows in both lungs.	Sudden Cardiac arrest on day 14. Died. Biopsy of the heart showed a few interstitial mononuclear inflammatory infiltrates, but no other substantial damage in the heart tissue.
	SARS	Ding et al.^4^	China, 2003	Case series (3)		There was myocardial stromal oedema. The endothelial cells of small veins were swollen and the vascular walls were oedematous and inﬁltrated by monocytes and lymphocytes. There were focal hyaline degeneration and lysis of cardiac muscle ﬁbres in one case.	
Tak-Sun et al.^6^		China 2003	Case report	A 64-year-old woman. 4-day history of ﬂuctuating fever up to 39∞C, dry cough with shortness of breath, and diarrhoea.	ECG: New T-wave inversion in the precordial leads. Laboratory: CK and cardiac troponin T levels were normal. TTE: normal left ventricular function with no regional wall motion abnormalities or pericardial effusion. Chest x-ray: right middle zone ground-glass haziness Angiography: normal coronary arteries. High-resolution CT scan (HRCT): presence of a pneumomediastinum.	Despite maximized medical treatment, she still complained of recurrent chest pain with reversible ECG changes of T-wave inversion in the precordial leads over the subsequent 6 days. Her chest pain gradually subsided with analgesics and the ECG showed normalization of T-waves in the precordial leads during the subsequent convalescent period.
Umapathi et al.^7^		Singapore, 2003	Case series (5)	**Case 1:** A 68-year-old woman, no risk factor with developed SARS that was complicated by respiratory failure, non-ST elevation acute myocardial infarction (AMI) **Case 2:** A 64-year-old woman. **Case 3:** A 54-year-old female with dyslipidemia. **Case 4:** A 63-year-old man, with diabetes hypertension and IHD. 2 weeks after admission he developed partial hemispheric syndrome **Case 5:** A 39-year-old man complicated with AMI, and cardiac arrhythmias.	**Case 1 :** Brain CT: Infarctions in the left posterior and middle cerebral artery (PCA, MCA) territories. TTE: EF 30%. **Case 2:** ECG: T inversion on all electrocardiograph leads. TTE: normal ejection fraction and ventricular wall motion. No clots or vegetations were noted. Brain CT: massive right MCA infarction with oedema and early hydrocephalus. **Case 3:** Brain CT: large infarctions in left PCA and bilateral MCA territories. No cardiac evaluation **Case 4:** Brain CT: left temporo-parietal infarction. TTE: Normal. **Case 5:** At autopsy: an infarction in the inferior lateral part of right occipital lobe was noted. Sterile vegetations in multiple cardiac valves, deep venous thrombosis and pulmonary embolism were detected. Maranteric endocarditis.	**Case 1:** Aspirin was started and LMWH discontinued. She remains aphasic and hemiplegic. IVIG for 16 days. She was weaned off the ventilator two months later. **Case 2:** LMWH for 2 days. Ventilation. Died 1 week later. **Case 3:** Her blood pressure dropped to systolic 60mmHg, but responded promptly to desmopression acetate (DDAVP), intravenous fluids and inotropic drugs. LMWH. IVIG. Died 2 days later. **Case 4:** No IVIG/No LMWH. Discharge **Case 5:** No IVIG/No LMWH. Died
Chong et al.^39^		Singapore, 2003	Case series (14)	Cases described in the study of Umapathiet al.	Cases described in the study of Umapathiet al.	20.5% had deep vein thrombosis, 11.4%, showed clinical evidence of pulmonary embolism, 15.9% had myocardial infarction, and 4.5% had a cerebrovascular accident.
MERS	Alhogbani et al.^40^	Saudia Arabia, 2016	Case report	A previously healthy 60-year-old man. 4-day history of fever, shortness of breath, cough with yellowish sputum, and left side chest pain, which was worsened by inspiration. Physical examination showed a body temperature of 38°C, blood pressure of 115/70 mm Hg, pulse rate of 120 beats per minute, and respiratory rate of 24 per minute. The jugular venous pressure was elevated.	Laboratory: elevated troponin-I level of 1.13 µg/L and an elevated pro-brain natriuretic peptide level of 6000 pg/ml, which increased to 8906 pg/ml on the second day. ECG: sinus tachycardia at a rate of 120 beats per minute and diffuse t-wave inversion. TTE: severe global LV systolic dysfunction and small pericardial effusion. CMR: LGe in favor of myocarditis.	During the second week of admission, the patient required hemodialysis because of acute renal failure, which improved after 4 weeks. He also required intubation for mechanical ventilation because of respiratory failure that continued for 6 weeks. 1 month rehabilitation. LV systolic function remained severely impaired on echocardiography performed 3 months after the first one. He was discharged home when his clinical condition was stable.
	Al-Abdallat et al.^41^	Jordan, 2014	Case series (12)*	-	-	Pericarditis, pericardial effusion, supraventricular tachycardia in one patient who died.

Abbreviations: AMI, acute myocardial infarction; CK-MB, Creatine Kinase MB; CMR, cardiac magnetic resonance imaging; COVID-19, Coronavirus Disease 2019; CT, computed tomography; ECG, electrocardiography; HscTnT, high sensitivity troponin T; IABP, intra-aortic balloon pump; IHD, ischemic heart disease; IVIG, Intravenous immunoglobulin; LGe, late gadolinium enhancement; LMWH, low molecular weight heparin; LVEF, left ventricle ejection fraction; MCA, middle cerebral artery; MERS, middle east respiratory syndrome; No, number; NT-proBNP, N-terminal pro-brain natriuretic peptide; PCA, posterior cerebral artery; RWMA, regional wall motion abnormality; SARS, severe acute respiratory syndrome; TTE, trans thoracic echocardiography; VAV, veno-arterial-venous; VV ECMO, veno-venous extracorporeal membrane oxygenation * 124 interviews with 12 confirmed MERS infection

### Original studies

#### Cardiovascular presentation

Ten COVID-19 manuscripts reported cardiac injury as the cardiovascular presentation of patients. However, only three manuscripts reported elevated creatine kinase levels in SARS patients without a significant increase in the MB isoform. Seven (one original study and six case studies) manuscripts reported electrocardiography changes and arrhythmias such as ventricular fibrillation and tachycardia as the presentation of patients infected with coronaviruses.

#### Cardiovascular complication

From the COVID-19 manuscripts, eight studies reported cardiac injury as a complication of the infection with incidence of 7.4%-77% according to the included patients’ disease severity. Heart failure was reported as the complication in four manuscripts with an incidence of 1%-49%. Regarding arrhythmias, only two studies reported them as the complication of COVID-19 infection with an incidence of 10.4%-60%. Studies related to SARS infection reported ischemic strokes, deep vein thrombosis (DVT), pulmonary embolism and cardiomegaly as cardiovascular complications of the infection.

#### Cardiovascular outcome

Most studies reported cardiac injury as predictors of disease severity and mortality. Some studies indicated that patients who had cardiac injury needed more intensive care unit hospitalization. Acute myocardial infarction, arrhythmias and heart failure were regarded as the cause of death in some patients.

### Case studies

Table 2 summarizes the different cases reported with COVID-19, SARS and MERS infection and their cardiovascular presentations, complications or outcomes. Overall, the cases included patients experiencing cardiogenic shock, myocarditis, ischemic stroke, myocardial infarction, DVT and pulmonary embolism.

## Discussion

As experience with COVID-19 grows, the management of cardiovascular problems has become one of the most important challenges in caring process of these patients. While the world is in the grip of the COVID-19 pandemics, we took a look back at SARS and MERS and tried to show differences and similarities between the three kind of Coronavirus infection in terms of cardiovascular manifestations and outcomes.

A similar pathogenesis has been suggested for three viruses. It has also been shown that the presence of cardiovascular comorbidities is accompanied with more severe illness and mortality in all three cases of Coronavirus infection.^1-43^ However, although the pandemics of COVID-19 has not ended, besides its reproductive number which is significantly higher in COVID-19 than SARS and MERS and their similarities such as more infection in adult and men, it seems that COVID19 compared to the other Coronavirus infections is more possible to manifest with a cardiovascular sign and symptom^3,4,10,41-42,44-45^ Interestingly, although it has been suggested that the three viruses may cause myocardial damage in victims, compared to SARS and COVID-19, the data regarding the cardiovascular manifestations of MERS were scarce and we could find limited reports regarding the presentation of MERS with cardiovascular manifestation.^40-41,46^

The acute cardiac injury or a myocarditis like syndrome which would be the most common cardiac involvement during Coronavirus infections has been more prevalently reported in those with COVID-19. The development of cardiovascular complications later in the course of the disease has also been reported more frequently in COVID-19.^2,3,10,13-14,47-48^

As far as we searched, the acute cardiac injury was not reported as initial presentation of SARS. Regarding the MERS, Alhogbani et al reported a 60-year-old man with fever, respiratory symptoms, left ventricular systolic dysfunction, pericardial effusion and elevated cardiac biomarkers with final diagnosis of acute myocarditis based on the cardiac MR findings.^40^ In a case series by Al-Abdallat et al a fatal case of MERS with pericarditis, pericardial and pleural effusions, and supraventricular tachycardia late in the course of illness has been reported.^41^

Thrombotic complications seem to be the point of similarity between the three Coronavirus infections; however, available data are limited in MERS compared to SARS and COVID-19. All forms of thrombotic complications including acute coronary syndromes, marantic valvular vegetations, deep vein thrombosis, pulmonary emboli, thrombosis in pulmonary veins, multi organ thrombosis, and thrombotic cerebrovascular accidents have been reported in both SARS and COVID-19. It is suggested that the thrombotic complications may be due to excessive inflammation, cytokine storm, platelet activation, endothelial dysfunction, and stasis. Also, the limited data in MERS suggests that the hematologic and coagulation disorders as the cause of the thrombotic complications in fatal cases.^3,7,29,39,42,46,49-51^

## Conclusion

In this study we tried to show the similarities and differences between the three types of Coronavirus. At the beginning the COVID-19 pandemic, the lessons from other Coronavirus epidemics suggested that the infection can be more severe and even fatal in those with cardiovascular co morbidities and a trigger for acute coronary syndromes, thrombotic complications and heart failure exacerbations; however, COVID-19 seems to be able to induce new cardiovascular pathologies and cardiovascular complications and appears to be a serious threat in addition to respiratory problems. It should also be clarified whether the nature of novel Coronavirus 2019 in terms of being more contagious and its spreading into different ethnicities and genetic backgrounds would be the cause of differences with its other ancestors. The long-term cardiovascular effects of COVID-19, along with the effect of future specific antiviral therapies are subject for further investigation. Further investigation is also needed to determine how patients with COVID-19 related cardiovascular complications should be followed in the long-term. Is there a possibility of recurrence in cases of myocarditis caused by the COVID-19? Will asymptomatic patients, with mild symptoms and people who have just suffered from respiratory problems without cardiovascular involvement, be at greater risk for heart diseases in the future?

## Ethical approval

The study was approved at the institutional research ethics committee with IR.RHC.REC.1399.005 ethical code.
